# Understanding Parental Representations Across the Perinatal Period: A Systematic Review of Empirical Findings and Clinical Implications

**DOI:** 10.3390/children12081051

**Published:** 2025-08-11

**Authors:** Renata Tambelli, Ludovica Del Proposto, Francesca Favieri

**Affiliations:** Department of Dynamic and Clinical Psuchology and Health Studies, “Sapienza” University of Rome, Via degli Apuli 1, 00185 Rome, Italy; delproposto.1885654@studenti.uniroma1.it (L.D.P.); francesca.favieri@uniroma1.it (F.F.)

**Keywords:** parental mental representation, reflective functioning, dyadic relationship, parent–infant interaction

## Abstract

**Highlights:**

**What are the main findings?**

- Parental mental representations (PMRs) develop during pregnancy and continue to evolve in the postnatal period.
- The current literature shows significant methodological heterogeneity and an underrepresentation of fathers and dyadic perspectives.

**What is the implication of the main finding?**

- Early identification of nonbalanced PMRs may help prevent relational difficulties and promote secure attachment.
- Future studies should adopt longitudinal, systemic, and culturally inclusive approaches to better capture the complexity of early parenthood and inform preventive mental health strategies.

**Abstract:**

**Background/Objectives**: Parental mental representations play a crucial role in shaping early parent–child relationships, particularly during the perinatal period. These internal models influence caregiving behaviors, emotional attunement, and the intergenerational transmission of attachment. The present systematic review aims to address this gap by examining the nature of both maternal and paternal mental representations in the perinatal period (involving pregnancy and the first postnatal time), with a particular emphasis on reflective functioning, and by outlining the variables that are influenced by these representations. **Methods**: Following PRISMA guidelines, eligible peer-reviewed studies were identified through a comprehensive literature search of major scientific databases (Scopus, Web of Science, PsychArticle/PsycInfo). Qualitative assessment and detailed description were carried out. **Results**: In total, 28 studies were selected and analyzed. Findings reveal that while representations tend to organize around shared psychological domains—such as expectations regarding the child, parental identity, and the anticipated relationship—there is significant heterogeneity in how these are conceptualized and measured across studies. Risk factors such as maternal depression, low social support, and adverse life experiences were consistently linked to disengaged or distorted representations, whereas balanced representations were associated with greater RF, emotional availability, and protective relational contexts. **Conclusions**: Overall, the review highlights the clinical relevance of assessing parental mental representations and RF during the perinatal period, suggesting that early, targeted interventions may enhance parental sensitivity and promote secure parent–infant bonds. Future research should adopt integrated theoretical models, include diverse family configurations, and evaluate the efficacy of preventive programs that support reflective and adaptive representations.

## 1. Introduction

Parental mental representations play a fundamental role in shaping the nature and quality of the parent–child relationship [1]. Since Bowlby’s introduction of the concept of internal working models [2], increasing attention has been paid to how parents mentally represent their children [3]. Pregnancy and generally the perinatal period are significant moments during which parents begin to form and then develop these representations of the child, initiating a psychological process that facilitates the child’s individuation and transition from an internal, imagined presence to a real, external one [4]. The perinatal period refers to a critical developmental window, encompassing the time from pregnancy through the first year postpartum, characterized by profound biopsychosocial change for both parents and infants, involving the emergence of parental identity, the establishment of early caregiving patterns, and the formation of foundational parent–child bonds [3,4,5].

This process aligns with Stern’s [5] distinction between two parallel worlds: the objective reality of the child and the subjective realm of parental mental representations. The latter encompasses not only real-life interactions but also—and perhaps more significantly—parents’ fantasies, hopes, fears, memories, and projections regarding their child’s future. Research has examined the content and structure of these representations, highlighting their importance in understanding how early caregiving experiences and prenatal perceptions shape future relational dynamics [1]. Evidence supports the idea that such representations influence both the quality of parent–infant relationships and child development outcomes [6].

A central component in the development of these representations is reflective functioning, defined as the capacity to understand one’s own and others’ behaviors and mental states [7]. Reflective functioning allows individuals to access emotional memories related to their own attachment and affective histories and relational experiences and to draw on these experiences to provide a secure relational base for their child. Given that attachment ‘experiences are strictly linked to regulation of affect, any framework aiming to understand attachment security and its intergenerational transmission must incorporate affective mentalization as the ability to reflect on emotions and how they relate to behaviors [8].

Reflective functioning, therefore, not only supports affect regulation and secure attachment, but also contributes directly to the formation and organization of parental mental representations. Through the ability to mentalize, parents can interpret their own and their child’s behaviors in terms of underlying thoughts and feelings, thereby enriching and structuring the internal image they hold of the child. This reflective stance allows for a more nuanced, flexible, and emotionally attuned representation, which is crucial for promoting sensitive caregiving. In this sense, reflective functioning serves as both a foundation and a dynamic regulator of parental representations, reinforcing their developmental significance from the prenatal period onward [1,9].

Among the most frequently investigated aspects of parental representations are expectations regarding one’s new identity as a parent, perceptions of the unborn child, anticipated lifestyle changes, and emotional reactions toward the future parent–child relationship. These representations often include imagined parenting characteristics (of oneself and one’s partner) and projected attributes of the child—both physical and psychological—especially after meaningful prenatal experiences such as feeling fetal movements or undergoing ultrasound scans [4].

Most studies on parental mental representations have primarily focused on mothers, with far less attention devoted to paternal representations. This is despite the importance of identifying similarities and differences in maternal and paternal attitudes and behaviors, and their respective influences on child development [10]. Research on fatherhood often emphasizes paternal well-being, attachment, and the quality of father–child interactions [11,12,13,14,15,16,17,18,19,20]. More recently, studies have also addressed prenatal bonding, psychological adjustment during the transition to fatherhood, and paternal expectations and representations [21,22,23].

In exploring parental and parent–child relationships, it is essential to consider how the transition to parenthood affects individual and dyadic identity, stress levels, and psychological adaptation [24]. Central to this process is the development of parental identity, emotional involvement in the pregnancy, and the evolving relationship with the unborn child. Equally important are the parent’s capacity to imagine the child, the quality of couple dynamics, and the ability to mentalize the child’s internal states—expressed through emotional availability, sensitivity, and responsiveness to imagined needs and feelings [25,26].

Over time, theoretical models of parental representations have evolved alongside changing conceptions of motherhood and fatherhood. These models also furnished the possible categorization of main parental mental representation, based on their coherence, emotional tone, and narrative structure. *Balanced/integrated representations* are characterized by coherence, emotional openness, and a view of the child as a separate individual with strengths and vulnerabilities, with personal temperament and behaviors. Parents with integrated representation express empathy and realistic expectations and can reflect on both positive and negative aspects of the relationship [1,4,6].

On the negative side of parental representation, which may be ascribed as *nonbalanced representation* [22], we can find *distorted representations*, characterized by confusion and rigidity, or excessive idealization, which may include contradictory statements or unrealistic expectations. We encounter *disengaged representations* that are expressively and emotionally flat, offer superficial description, and are lacking in detail, often being marked by emotional detachment or minimization of the importance of the parent–child bond. As reported by Vreeswijk’s review [6], these typologies are clinically relevant, as they have been shown to predict different patterns of early caregiving behavior and child attachment.

However, despite a growing body of empirical research, a comprehensive synthesis that integrates findings on both maternal and paternal representations is still lacking. Understanding parental mental representations is not only essential for developmental research but also has practical implications for the early identification of parenting difficulties and for designing targeted interventions aimed at enhancing parental sensitivity and mentalization, thereby fostering secure attachment and emotional well-being in children [27]. The present systematic review aims to address this gap by examining the nature of both maternal and paternal mental representations in perinatal period, including studies focusing on both the prenatal stage (from conception to childbirth) and the postnatal stage (up to 12 months postpartum), with a particular emphasis on reflective functioning, and by outlining the variables that are influenced by these representations. This approach allows us to capture the continuity and transformation of parental mental representations across a developmental continuum that is essential for understanding early relational health.

## 2. Materials and Methods

### 2.1. Research Strategies

This systematic review was conducted according to the Preferred Reporting Items for Systematic Reviews and Meta-Analyses (PRISMA) guidelines for meta-analysis [28]. The research was conducted in Scopus, PsycArticles and PsycInfo databases using keywords related to parenting and maternal and paternal mental representations. Specific scripts and databases are reported in Table 1. All original papers published in peer-reviewed journals up to 15 May 2025 were searched, without any date filtering. Our efforts searching the databases and saving records for systematic review were conducted between 14 May 2025 and 15 May 2025.

### 2.2. Eligibility Criteria

Studies included in this systematic review examined parents’ mental representations. Specific inclusion criteria were (a) studies involving biological parents; (b) studies in which interviews were administered during pregnancy and/or (c) in the postnatal period; and (d) studies employing instruments specifically developed and/or validated for the assessment of parental mental representations, in both their balanced and integrated and nonbalanced (i.e., poor, distorted and disengaged) expression. To guarantee an adequate level of control and reduce heterogeneity, studies were excluded if they (a) involved nonbiological parents (e.g., adoptive parents) or parents who had undergone (b) Medically Assisted Reproduction or In Vitro Fertilization; (c) focused on high-risk families (i.e., in cases of addiction, or physical/mental health diagnosis affecting the parent or infant); or (d) involved adolescent parents. Finally (e) reviews, theoretical papers, or conference abstracts were excluded, as were (f) studies published in languages other than English or Italian.

### 2.3. Data Collection Process

Data selection and subsequent extraction were carried out by identifying studies through title and abstract screening. From the selected articles, information was extracted regarding participants (gender, age, sample size), the assessment period (gestational month, postpartum period), the instruments used to assess parental mental representations, the dimensions investigated, the psychological variables considered, and finally the main findings.

### 2.4. Quality Assessment Methods

The quality of each paper included in the final stage of the systematic review was explored. This assessment was conducted through the adaptation of the Higgins’ tool for risk of bias [29,30], structured to assess bias for the following domains: (I) *sampling bias*: the inclusion of small, unbalanced, or incomplete datasets, often drawn from non-representative populations (e.g., specific cultural, ethnic, or clinical groups), limiting the applicability of the findings to the general population; (II) *method bias*: the frequent use of non-standardized, non-validated, or ad hoc instruments—such as self-developed questionnaires or interviews lacking sufficient description—raises concerns about the reliability and replicability of the findings; (III) *results bias:* unclear results, or data not clearly explained in the results section, qualitative analyses or lack of disclosure of the effect size; (IV) *discussion bias*: minimal elaboration on the main focus of the study, weakening the overall interpretative value.

**Figure 1:**
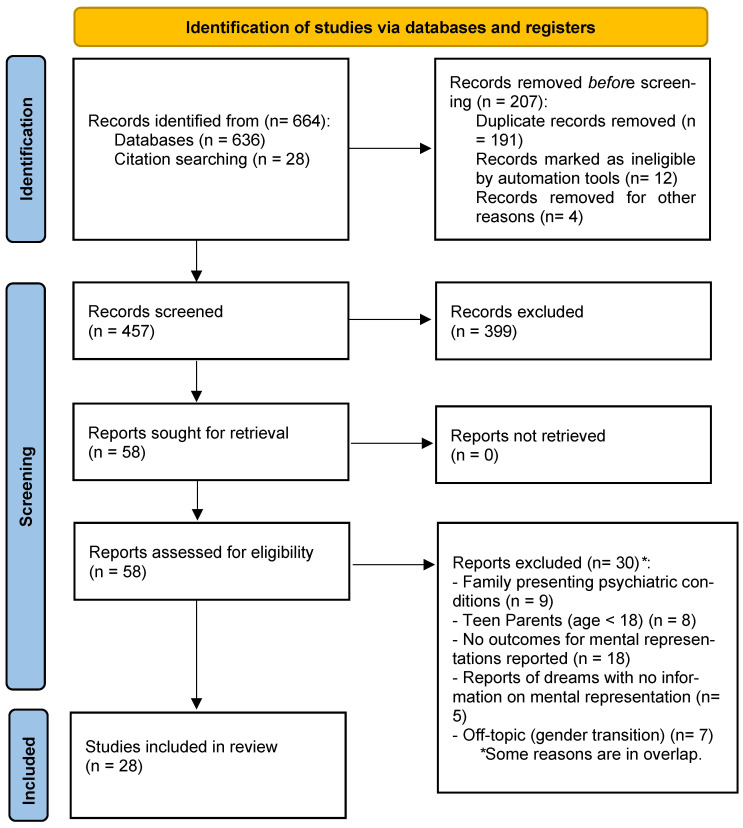
PRISMA flow diagram [28].

Each domain was evaluated according to the following code: no or low risk = 0; medium risk = 1; high risk = 2. Two independent reviewers (F.F., L.D.P.) evaluate each full text according to the indication reported in Table 2. Discordance was solved with the mediation of a third supervisor (R.T.). The global quality of the review, considering the percentage of the studies with low–medium–high risk of bias, was reported in a graphical summary (Figure 1).

## 3. Results

### 3.1. Study Selection and Inclusion

Flow diagrams report the search process and inclusion of the records. After the first screening phase, 86 articles were eligible for full-text checking. At the end of the process, 58 articles were excluded (see Figure 1 for detailed information); in the final sample, 28 studies were included in this systematic work.

### 3.2. Quality Assessment

The qualitative analysis used to identify the risk of bias for each study in the following domains—(i) sampling, (ii) methodology, (iii) results, and (iv) discussion—highlighted that 96% of the studies reported a low risk of error, while 3% (1/28) reported a moderate risk of error, indicating overall good quality among the included studies, with no studies presenting a high risk of error. The analysis of the domains revealed that the moderate risk of bias was primarily due to issues in the sampling and methodology domains. Many authors did not sufficiently clarify the inclusion criteria for participants (e.g., [31]), while others included small or heterogeneous samples (e.g., [4]) or did not explicitly describe the characteristics of the participants (e.g., [32]).

### 3.3. Characteristics of the Included Studies

The total sample of the review included approximately 8848 parents, with a range of sample sizes from 23 [4] to 1646 [31]. The large number of studies focused on mothers (66.54%), while studies including exclusively fathers were 17.38%, and the couples of parents were included in 16.07% of the studies. The age of parents ranged from 16 [33] to 50 years [34], despite not all these studies clearly reported this information (see Table 2 and Table 3 for details).

Overall, 19 out 28 studies adopted structured and semi-structured interviews [4,22,23,32,34,35,36,37,38,39,40,41,42,43,44,45]. In particular, 16 studies [4,22,23,32,34,35,36,37,38,39,41,43,44,45,46,47] used validated tools, such as the Working Model of the Child Interview (WMCI) [48], the Interview of Maternal Representations during Pregnancy [Intervista sulle rappresentazioni materne in gravidanza (IRMAG)] [4], the Clinical Interview for Parents During Pregnancy [Colloquio per Genitori in Gravidanza (CGG)] [49,50], the Interview on Maternal Representations [51], and the Interview of Paternal Representations during Pregnancy [Intervista sulle rappresentazioni paterne in gravidanza] [36]. Meanwhile, Delmore-Ko and colleagues [40], Ilicali & Fisek [42], and Madsen and colleagues [52] adopted ad hoc tools (ie., The Prenatal Interview, Maternal Representations Interview, and Father Attachment Interview).

Nine studies [31,33,53,54,55,56,57,58,59] used exclusively self-report instruments. Specifically, they used the What Being a Parent of a New Baby is Like-Revised (WPL-R) [60], the Subjective Family Picture Test, The Pie [61], the Infant Characteristics Questionnaire (ICQ) [62], the Childbearing Attitudes Questionnaire [63], the Maternal Self-Definition Questionnaire (MSDQ) [64], the Representations of Unborn Baby (RUB-M), and the Child Concept Questionnaire [65]. Coleman and colleagues [33] (1999) also used an ad hoc tool, the Prenatal Maternal Expectations Scale (PMES), while Gress-Smith et al. [54] and Pajulo et al. [56] used exclusively self-report tools created ad hoc, specifically the Prenatal Experiences Scale for Mexican Americans (PESMA), an adapted version of the PMES, and the Prenatal Parental Reflective Functioning Questionnaire (PRF-Q).

In general, all studies included in the systematic review investigated both maternal and paternal mental representations before birth, either longitudinally [23,31,33,34,35,39,40,41,45,46,47,52,53,57,58,59] to assess changes over time (e.g., pre-postpartum), or by focusing exclusively on representations during the prenatal phase [4,22,32,36,37,42,43,44,54,55,56].

Several studies explored characteristics of both maternal and paternal mental representations, while others considered their relationship with other constructs. The further paragraphs furnish deepen insight into the main outcomes of the studies.

#### 3.3.1. Parental Mental Representations

During pregnancy, parents develop mental representations of themselves and their unborn child, which are essential for bonding and caregiving [4]. These representations evolve across pregnancy and peak in complexity around the seventh month [43]. By this stage, most mothers have a distinct maternal identity, though integration between self-as-woman and self-as-mother is still ongoing. Fathers also develop representational models, shaped in part by social referencing, i.e., the degree to which their parental identity is influenced by others’ expectations [36].

Social referencing correlates negatively with flexibility and emotional involvement in both mothers and fathers [4,36]. Specifically, among mothers, social referencing is negatively correlated with openness to change, suggesting that socially anchored representations tend to be less flexible and adaptive [4]. Similarly, in fathers, social referencing is inversely associated with richness of perception, emotional engagement, openness to change, and the emergence of fantasies [36].

Representations of the unborn child, for mothers, often reflect the partner’s traits while shifts postnatally tend to align child representations with the mother’s self-image [4,42]. Over time, representations of self, child, and partner differentiate, reflecting the processes of identity formation and individuation [45]. In the early postnatal period, studies report gradual increases in maternal self-confidence, information-seeking related to the child, and identification with pregnancy. These trends suggest that pregnancy supports a growing sense of independence, embodiment, and the anticipation of fulfillment in motherhood [58]. Simultaneously, decreases were observed in maternal worries, sexual interest, and negative feelings related to infant care—such as discomfort with public breastfeeding or irritation at the disorder linked to childrearing [58].

For fathers, self-representations are strongly correlated with child representations across multiple dimensions [36]. Fathers with integrated representations thus hold a mental image of the child that is richer, more emotionally involved, flexible, coherent, and imbued with fantasy. Like maternal trends, integrated/balanced representations were the most common, while restricted/disinvested and non-integrated/ambivalent types were less frequent [36,37].

Maternal representations appear to remain largely stable over time, especially among those with balanced representations [47]. A similar pattern is found in fathers, those with nonbalanced representations tend to show greater changes after birth, while balanced ones remain more stable [22].

#### 3.3.2. Parental Mental Representations and Reflective Functioning

Parental reflective functioning (PRF) is closely related to representational quality. Low prenatal PRF is associated with disengaged representations, while high postnatal PRF predicts balanced ones [32,35,44,56]. However, inconsistencies emerge across the studies, with some individuals showing high PRF alongside distorted representations [35].

Mothers consistently show higher reflective functioning than fathers across domains involving the child, couple relationship, and their own parents [44]. Fathers tend to reflect less on mental states and their implications for behavior. Both parents demonstrate greater reflective capacity when discussing the child rather than their past or their partner.

First-time mothers outperform experienced mothers and fathers in considering mental states and acknowledging their variability, though no gender difference appears in recognizing the opacity of mental states [56]. Finally, paternal reflective functioning is linked to caregiving style: low PRF is associated with instrumental or detached involvement, while moderate-to-high PRF predicts expressive and emotionally engaged parenting [32].

#### 3.3.3. Parental Mental Representations and Risk Factors

Four studies have investigated parental mental representations in at-risk and non-at-risk samples [31,34,37,55]. Ammaniti and colleagues [37] found that at-risk mothers—exposed to depression, low socioeconomic status, abuse, and poor support—were less likely to develop integrated/balanced representations and scored lower on dimensions such as coherence and openness to change. Pajulo et al. [55] similarly reported that at-risk mothers had less functional representations across various domains, especially regarding their partner, reflecting relational difficulties. Rusanen et al. [31] highlighted that those supportive relationships fostered more positive prenatal expectations, whereas depressive symptoms—identified as the strongest risk factor—were linked to more negative caregiving representations. Interestingly, higher maternal education and younger age were also associated with more negative or inconsistent expectations. Vreeswijk and colleagues [34] found that balanced representations were more frequent in mothers postnatally, while fathers showed increased distorted representations after birth. Maternal representations were more sensitive to prenatal risk, and families in which both parents held balanced representations reported fewer risk factors overall [22,23,34].

#### 3.3.4. Parental Representations and Child Attachment

Three studies examined the association between parental representations and subsequent child attachment, using the Strange Situation Procedure (SSP; [66]) [39,41,46]. Benoit et al. [39], using the Working Model of the Child Interview (WMCI; [48]), found that balanced representations during pregnancy were strongly associated with secure attachment at 11 months. This predictive continuity was evident for balanced and distorted categories, but not for disengaged ones. Huth-Bocks et al. [41] further confirmed this association and demonstrated that maternal representations were more strongly influenced by prenatal risk factors—such as demographic risks and domestic violence—than the attachment outcome itself. Maternal self-representations were closely linked to child representations, and lower levels of prenatal social support were associated with less balanced maternal representations. In contrast, greater prenatal support predicted postnatal support, which was associated with more secure attachment. Similarly, balanced representations were consistently linked to secure attachment at one year of age. Tambelli et al. [46], using IRMAG and IRPAG interviews [4,36], explored how maternal and paternal prenatal representations influence emotional availability and attachment. For mothers, prenatal representations had a direct predictive effect on both attachment and emotional availability, whereas emotional availability alone did not directly affect attachment style. In contrast, among fathers, prenatal representations predicted emotional availability, which in turn influenced infant attachment. However, neither paternal representations nor emotional availability directly predicted attachment classification, highlighting distinct pathways in maternal and paternal contributions to early attachment formation.

#### 3.3.5. Parental Mental Representations and Postnatal Experience

Five studies examined how parents’ prenatal mental representations relate to their postnatal psychological adjustment and experience of parenting [40,53,54,57,59].

Delmore-Ko et al. [40], in their longitudinal exploration, identified distinct parental attitudes—enthusiasm, anxiety, coping, uncertainty, and socialization—with mothers generally reporting higher stress and depression, and fathers experiencing an higher reduction in marital satisfaction in the months after the birth, referring to the quality of the relationship with the partner, although this dissatisfaction is reported also by the mothers. Both parents anticipated challenges despite some positive expectations. Flykt et al. [53] found that high prenatal expectations for emotional closeness and autonomy predicted lower parental stress after birth, although some moderate expectations also influenced stress levels differently for mothers and fathers. Mothers’ expectations about their own relationship with the child improved postpartum, while views on their partner’s involvement declined. Gress-Smith et al. [54] showed that family and paternal support, along with maternal role fulfillment, were linked to fewer depressive symptoms, particularly among younger, married, and more educated mothers. Paternal well-being was not clearly associated with prenatal child representations. Pearce and Ayers [57] and others confirmed that both prenatal expectations and postnatal evaluations affect the mother–infant bond, with postnatal perceptions having a stronger influence. Negative prenatal expectations increased the risk of weaker bonding. Thun-Hohenstein and colleagues [59] found no direct prenatal predictors of maternal competence, though child desirability related to better mother–infant interaction. Maternal competence was higher in younger, more educated mothers and those with male infants.

**Table 2:** PICOS’ table of the studies included in the systematic review.

**Author (Year)**	**Sample Size/Parent**	**Mean Age (M)/Age Range (AR)**	**Assessment Period**	**Assessment Tool for Mental Representations**	**Investigated Dimensions (Outcomes)**	**Main Findings**
Alismail et al., 2021 [35]	47 mothers	M = 25.74	Third trimester to 7 months postpartum	WMCI (PP); PI-R; PDI (PP)	Associations between maternal reflective functioning (prenatal and postnatal) and maternal representations at 7 months postpartum.	Representations categorized as distorted (38.3%), balanced (36.2%), and disengaged (25.5%). Lower prenatal MRF levels were associated with disengaged representations. Higher MRF was linked to balanced representations. No significant difference in MRF scores between distorted and disengaged groups. Some inconsistencies found (e.g., high MRF with distorted representations).
Ammaniti et al., 1992 [4]	23 primiparous mothers	M = 29 AR = 20–34 years	28–32 weeks of pregnancy	IRMAG	Content and structure of maternal representations.	Clear differentiation between self as mother and baby representations. Strong positive correlations across matched dimensions of self and baby representations. Social dependency inversely related to openness to change. Baby’s characteristics perceived as more similar to the father than to the mother. Mothers emphasized differentiation from their own mothers.
Ammaniti et al., 2006 [36]	130 fathers	M = 33 AR = 23–52 years	28–32 weeks of pregnancy	IRPAG	Organization of paternal representations and parenting styles.	Positive correlations between self and baby representations. Correlation between richness of perceptions, openness to change, emotional engagement, and the emergence of fantasies Three representation types: Integrated (*n* = 72), Restricted/Disinvested (*n* = 44), Ambivalent/Unintegrated (*n* = 14)
Ammaniti et al., 2013 [37]	666 mothers (411 low-risk; 255 at psychosocial/depressive risk)	M = 31.67 AR= 23–43 years	Mid-6th to mid-7th month of pregnancy	IRMAG	Prevalence of representation categories in risk vs. non-risk groups.	Three representation categories identified: Integrated, Restricted/Disinvested, Ambivalent/Unintegrated. Risk-group mothers showed lower scores across most dimensions and higher social dependency. Self-as-mother representations more articulated than representations of the child.
Bailes et al., 2024 [38]	297 mothers	M = 31.17 AR: 21–44 years	Second trimester to 6 months postpartum	WMCI	Link between perception richness, pregnancy acceptance, intention, sensitivity, and warmth.	Greater pregnancy intentionality predicted higher acceptance and perception richness in the third trimester. These elements mediated higher caregiver sensitivity and warmth in early interactions.
Benoit et al., 1997 [39]	96 mothers	M = 29.17 AR = 20–39 years	Third trimester to 12 months postpartum	WMCI	Predictive validity and stability of maternal representations; associations with attachment classification.	Balanced WMCI representations were strongly associated with secure attachment (Strange Situation). Prenatal WMCI categories remained stable at 11 months, particularly balanced and distorted types. No stability for disengaged representations. Significant concordance between prenatal WMCI and infant attachment classifications
Coleman et al., 1999 [33]	31 mothers	M = 25 AR = 16–32 years	Third trimester to 3 weeks postpartum	PMES; WPL-R	Prenatal expectations and postnatal maternal attitudes.	Higher PMES scores predicted more positive postnatal maternal attitudes (WPL-R). Low PMES scores correlated with negative postnatal adjustment. Moderate prenatal expectations did not predict more positive postnatal attitudes.
Delmore-Ko et al., 2000 [40]	59 first-time parent couples	M (mothers) = 27.3 AR (mothers) = 18–40 years M (fathers) = 30.1 AR (fathers) = 19–48	Third trimester to 18 months postpartum	Prenatal Interview	Future parenting expectations and relationship changes.	Four maternal themes: Enthusiasm, Anxiety, Coping, Uncertainty. Five paternal themes: same as mothers plus Socialization. Couples showed the highest adjustment in the third trimester. Marital satisfaction declined after parenthood for both parents.
Flykt et al., 2011 [53]	378 parent couples	M (mothers) = 33.3 M (fathers) = 34.2	Second trimester to 12 months postpartum	Subjective Family Picture Test (child-related items)	Parental expectations and parenting stress.	High prenatal expectations of intimacy and autonomy with the baby associated with lower parenting stress at 2 and 12 months. Maternal expectations about emotional intimacy with the baby predicted lower stress more than paternal ones. Moderate paternal expectations of autonomy predicted lower stress at 2 months. Mothers’ expectations positively changed postnatally; paternal expectations remained mostly stable.
Gress-Smith at al., 2013 [54]	210 mothers	M = 27.4	Third trimester	PESMA	Maternal expectations and their correlates.	Higher expectations of maternal role fulfillment and partner support were linked to lower depressive symptoms and higher perceived support. Married/cohabiting women had higher expectations of partner support. Higher expectations of satisfaction from motherhood were associated with readiness to become a mother, greater financial difficulty, and previous childcare experience. High family support expectations were linked to younger age, fewer children, higher education, and financial challenges
Huth-Bocks et al., 2004 [41]	206 mothers	M = 25.4 AR = 18–40 years	Third trimester to 1 year postpartum	WMCI	Predictive value of maternal representations for attachment quality.	Strong correlations between representations of the child and of the self as mother. Negative childhood attachment experiences linked to lower prenatal support and less secure prenatal representations. More prenatal risk factors correlated with less secure representations. Secure prenatal representations predicted more secure mother–infant attachment.
Ilicali, Fisek, 2004 [42]	45 primiparous mothers	M = 22.96	4–8th month of pregnancy to 7 months postpartum	Entretien R1, MRI	Maternal representations before and after birth.	Pregnant women often identified their future children with their husbands; postpartum, this shifted toward self-identification. Mothers sought differentiation from their own mothers, favoring idealized maternal roles. Postpartum narratives were more coherent, flexible, and richer than prenatal ones.
Innamorati et al., 2010 [43]	162 mothers divided into: Early pregnancy (EP, *n* = 55), Mid pregnancy (MP, *n* = 51), Late pregnancy (LP, *n* = 55)	M (EP) = 30.3 M (MP) = 30.2 M (LP) = 31.8	Varies by group (pre-5th month, 6–7th month, 8–9th month)	The Breakfast Inteview MIPS	Evolution of the maternal constellation.	Mid-pregnancy (MP) mothers scored highest on all four themes (growth concern, affective involvement, support, identity transformation). Maternal constellation peaked in richness and specificity during the 6–7th month.
Lis et al., 2000 [44]	112 first-time parent couples	M (mothers) = 24 M (fathers) = 28	7th month of pregnancy	CGG Reflective Functioning Scale	Parenting styles and reflective function.	Most common score was 3 on reflective function. Mothers scored higher than fathers. Reflective function was lower in the “child” area than in others.
Lis et al., 2004 [32]	112 fathers	M = 28	7th month of pregnancy	CGG Reflective Functioning Scale	Parenting styles and reflective function.	Low reflective functioning predicted instrumental or observer parenting styles. Moderate-to-high reflective functioning predicted expressive style.
Madsen et al., 2007 [52]	41 fatehrs	-	6th month of pregnancy to 5 months postpartum	Father Attachment Interview	Paternal reflective functioning and caregiving representations.	Fathers’ ability to reflect on their child’s mental states was associated with their own caregiving models (especially with maternal caregiving). Reflective functioning was predicted by internalized models based on closeness, compassion, and understanding.
Pajulo et al., 2001 [55]	380 mothers (84 at risk)	M = 27.5	3rd to 8th month of pregnancy	IRMAG	Content of maternal representations and risk factors.	At-risk mothers showed lower scores in representations of the baby, self as mother and woman, their partner, and their own mother. Positive representations of the baby were more similar across risk and non-risk groups.
Pajulo et al., 2015 [56]	Pilot: 124 mothers, 82 fathers|Cohort: 600 mothers, 600 fathers	M (mothers in cohort) = 30.1 M (fathers in cohort) = 32.1	20–32 weeks of gestation	P-PRFQ PI	Development of a prenatal reflective functioning questionnaire.	Mothers scored higher in considering mental states and relational flexibility. Both mothers and fathers showed similar levels of perceived opacity of mental states. Maternal reflective functioning correlated with PI scores.
Pearce and Ayers, 2004 [57]	51 mothers	M = 31	Week 39 of gestation to 3 weeks postpartum	ICQ, Mother–Baby Self-Rating Scale	Stability of maternal perception and its relation to bonding.	Expectations during pregnancy correlated with postnatal perceptions and mother–infant bonding. No association found between expectation–evaluation mismatch and poor bonding or postnatal distress. Negative expectations were associated with higher risk of poor bonding.
Ruble et al., 1990 [58]	Cross-sectional: 667 mothers|Longitudinal: 48 mothers	M (cross-sectional) = 29 AR (cross-sectional) = 18–42 M (longitudinal) = 29 AR (longitudinal) = 18–37	Cross-sectional: 2 years pre-conception to 3 months postpartum Longitudinal: 9th month pregnancy to 16 months postpartum	CAQ MSDQ	Changes in self-definition.	Self-perceptions were stable by the 9th month. Postpartum women felt more involved and protective. Across phases: increases in maternal self-confidence and information-seeking; decreases in maternal concerns and sexual interest.
Rusanen et al., 2018 [31]	1646 mothers	M = 30.2	32nd week of pregnancy to 24 months postpartum	RUB-M	Moderating factors in the development of maternal representations.	Positive expectations about bonding, caregiving, and routines were linked to better family atmosphere. Stress, anxiety, and depressive symptoms were associated with more negative expectations. Higher maternal education was linked to fewer positive expectations and more caregiving concerns.
Tambelli et al., 2020 [46]	50 first-time parent couples	M (mothers) = 33.88 M (fathers) = 36.90	7th month of pregnancy to 18 months postpartum	IRMAG-R IRPAG-R EAS Strange Situation	Prenatal representations, emotional availability, and infant attachment.	Strong links between prenatal representations, emotional availability, and attachment categories. For mothers, prenatal representations directly influenced attachment. For fathers, emotional availability mediated the link with attachment quality.
Theran et al., 2005 [47]	180 mothers	M = 25 AR = 18–40 years	Third trimester to 1 year postpartum	WMCI	Stability and change in maternal mental representations	71% showed stable representations over time. Balanced representations were most stable. Nonbalanced types were associated with low income, single parenting, and prenatal abuse.
Thun-Hohenstein et al., 2008 [59]	73 mothers	M = 29.2 AR = 19–39 years	Third trimester to 3 months postpartum	CCQ	Predictive value of prenatal representations on early mother–infant interaction.	No link between maternal competence and predictive variables. Desirability of unborn child predicted eye contact in still-face procedure. Readiness for interaction was linked to prenatal relational representations. Higher parental competence found in mothers of boys, younger mothers, and those with more education.
Vizziello et al., 1993 [45]	42 mothers	M = 29 AD = 20–35 years	7th month of pregnancy to 4 months postpartum	Interview on Maternal Representations	Role of maternal representations in early bonding and clinical assessment.	Four representation themes: Desire-driven, Defensive, Fear-based, Disorganized. Representations of self as woman and of partner were stable; those of self as mother and of the baby were less stable. Integration process occurred between maternal and female identity.
Vreeswijk et al., 2014 [22]	243 fathers	M = 34.01 AD = 27–49 years	26th week of pregnancy	WMCI PAAS	Meaning of the unborn child for the father.	Fathers were more often in the “disengaged” category, mothers in “balanced”. Prenatal attachment quality was negatively associated with depressive and anxious traits. Younger age, first-time fatherhood, and higher education linked to better prenatal attachment.
Vreeswijk et al., 2014 [23]	217 fathers	M = 34.11 Range = 22.31–49.60	Week 26 of pregnancy to 6 months postpartum	WMCI	Stability and predictors of paternal representations.	Over half had nonbalanced representations prenatally. Fathers with nonbalanced prenatal representations more likely to change postnatally. First-time fathers had more balanced prenatal representations. No significant effect of paternal mental well-being on representations. Conscientious and agreeable fathers had more balanced representations across time
Vreeswijk et al., 2015 [34]	294 mothers 225 fathers	M (mothers) = 31.60 AR (mothers) = 17–42 M (fathers) = 34.09 AR (fathers) = 22–49	26th week of pregnancy to 6 months postpartum	WMCI	Prenatal risk factors and stability of representations.	Mothers were more likely to develop balanced and less likely disengaged representations postpartum. Fathers showed similar patterns but increased distorted representations after birth. Maternal prenatal risk was linked to distorted representations; no such link in fathers. Disengaged prenatal mothers remained disengaged; balanced fathers remained balanced postnatally.

WMCI = Working Model of the Child Interview; PDI = Parent Development Interview; IRMAG = Interview on Maternal Representations in Pregnancy (Intervista sulle Rappresentazioni Materne in Gravidanza); IRPAG = Interview on Patern Representations in Pregnancy (Intervista sulle Rappresentazioni Paterne in Gravidanza); PMES = Prenatal Maternal Expectations; WPL-R = What Being the Parent of a New Baby is Like- Revised; CGG = Consultation for Expectant Parents (Colloquio per Genitori in Gravidanza); P-PRFQ = Prenatal Parental Reflective Functioning Questionnaire; PI = Pregnancy Interview; ICQ = Infant Characteristics Questionnaires; CAQ = Childbearing Attitudes Questionnaire; MSDQ = Maternal Self-Definition Questionnaire; RUB-M = Representations of Unborn Baby; EAS = Emotional Availability Scales; PAAS = Paternal Antenatal Attachment Scale.

**Table 3:** Details of demographic variables and risk factors reported by the studies.

**Author (Year)**	**Demographic Data and Risk Factors**	**Main Characteristics**
Alismail et al., 2021 [35]	Marital Status Race/Ethnicity Education Income (yearly)	51% single 78.7% black 27.7% high school; 44.7% college 70% < 30,000 $
Ammaniti et al., 1992 [4]	n/a	n/a
Ammaniti et al., 2006 [36]	n/a	n/a
Ammaniti et al., 2013 [37]	Depressive risk Psychosocial Risk *Assessed* via *CES-D*	Low risk = 411 mothers High risk = 255 mothers
Bailes et al., 2024 [38]	Marital Status Race/Ethnicity Education Income (yearly)	15% single 85% Caucasian 75% bachelor’s degree or higher median 90,000–150,000 $
Benoit et al., 1997 [39]	Marital Status Race/Ethnicity Education Income	5% single Caucasian Range = 9–27 years Upper-middle-class background
Coleman et al., 1999 [33]	Marital Status Education Employing	16% single 42% bachelor’s degree or higher Full time
Delmore-Ko et al., 2000 [40]	Marital Status Education Employing Psychological Risk	Average of marriage = 4.2 35% bachelor’s degree or higher Both parents = full time Stress; depressive symptoms; self-esteem; marital adjustment
Flykt et al., 2011 [53]	n/a	n/a
Gress-Smith at al., 2013 [54]	Marital Status Race/Ethnicity Education Economic hardship Depressive Symptoms Social support	7% divorced; 15% single 86% Mexican 5.7% bachelor’s degree or higher Average = −0.03 Range = 0–25; average = 5.4 Middle-high
Huth-Bocks et al., 2004 [41]	Marital Status Race/Ethnicity Education Income (monthly) Domestic violence in life Domestic violence in pregnancy	50% single 63% Caucasian 13% bachelor’s degree or higher Range = 0–$9500; Average = $1451 75% 44%
Ilicali, Fisek, 2004 [42]	Marital Status Education	Average = 2 years Average = 11 years
Innamorati et al., 2010 [43]	Race/Ethnicity Education	100% Italian 17% middle school or lower
Lis et al., 2000 [44]	Socioeconomic status *Assessed* via *Four-Factor Index*	Medium socioeconomic level
Lis et al., 2004 [32]	Education Socioeconomic status *Assessed* via *Four-Factor Index*	52% high school Medium socioeconomic level
Madsen et al., 2007 [52]	n/a	n/a
Pajulo et al., 2001 [55]	Risk conditions group screening for any of the variables considered	Depressive symptoms; Risk for addiction; Social Environmental difficulties; Lack for social support.
Pajulo et al., 2015 [56]	Marital Status Education Income (monthly)	98% married 68% high levels 24% < 2000 €
Pearce and Ayers, 2004 [57]	Marital Status Race/Ethnicity Education Socioeconomic status	100% in a relationship 76% European 27% bachelor’s degree or higher Class 2–3 = 88%
Ruble et al., 1990 [58]	Race/Ethnicity Education	98% Caucasian 70% bachelor’s degree or higher
Rusanen et al., 2018 [31]	Education Income (monthly) Psychological Screening	33.4% bachelor’s degree or higher 74% < 2000 € Anxiety; Depressive symptoms; Family Atmosphere.
Tambelli et al., 2020 [46]	Psychological Screening	Anxiety; Depressive symptoms; Emotional Availability.
Theran et al., 2005 [47]	Psychological Screening	Depressive symptoms
Thun-Hohenstein et al., 2008 [59]	Education Psychological Screening	44% Low Depressive symptoms
Vizziello et al., 1993 [45]	n/a	n/a
Vreeswijk et al., 2014 [22]	Education Employing Psychological Screening	64.2% bachelor’s degree or higher 96.6% Anxiety; Depressive symptoms
Vreeswijk et al., 2014 [23]	Education Employing	65.4% > 9 years 96.3%
Vreeswijk et al., 2015 [34]	Education Employing First child	64% > 9 87% of mothers; 97.9% of fathers 51.6%

CES-D = *Center for Epidemiological Studies-Depression Scale*.

## 4. Discussion

This systematic review synthesized the current state of knowledge on parental mental representations in the perinatal period and their role in influencing parent–infant interaction and positive child development. The qualitative synthesis offered critical insights into their structure, determinants, and implications for early relational development. Overall, parental mental representation appears to be shaped by a complex interplay of individual, relational, and contextual factors, with important implications for child development and clinical practice. Also, significant themes for future research and intervention programs emerge.

An initial result that clearly emerged is the heterogeneity in methods for the exploration of the representations that may influence the outcomes, reporting inconsistent findings. This lack of consistency ranged from divergent constructs (e.g., representational balance, coherence, emotional tone) to the use of different tools (e.g., IRMAG/IRPAG, WMCI, PAI, PDI-R2), reducing the comparability of the studies. While some studies highlighted the predominance of integrated or balanced paternal representations [36,37], others found a higher prevalence of disengaged representations [22,23]. However, these discrepancies would reflect cultural differences and emphasize the need to consider country-specific parenting norms and values, as well as further explore how for mothers and fathers’ parent reflective functions may affect the representation of child, and future parent–infant relationships. Through this work, although parental mental representations converged towards common and general dimensions, ascribed to thinking about parents’ experience (future or current ones) and the physical, mental, and lifestyle characteristics of their child (or future child), a non-univocal approach emerged. As suggested by some authors, the range of representational qualities may differ in depth, coherence, and affective tone across the different coding systems. In this systematic review, the need for a unified theoretical framework emerges, especially in order to provide strong evidence for clinical applicability [67,68].

Interestingly, multiple risk and protective factors influencing the development of prenatal mental representations and the future parent–child relationship and child well-being in terms of healthy development emerged via the studies. Risk factors include depressive symptoms, low education, and socioeconomic status, family psychiatric history, significant life stressors (e.g., losses and separations), and a lack of social support. Individuals with such risk factors in their medical or psychosocial history often encounter difficulties in developing prenatal representations of the child with an impact on the quality of the future dyadic relationship [31,34,37,41,55]. For instance, in the study by Rusanen et al. [31], mothers with significant psychosocial risks were more likely to express distorted prenatal representations, characterized by fear, emotional disconnection, and idealization. Similarly, Ammaniti et al. [37] found that a lack of emotional support and unresolved losses in family history were associated with disengaged parental narratives, especially in fathers. Longitudinal evidence suggested that, in this frame of risk, more challenges in establishing secure parent–infant bonding are reported [26,69].

On the other hand, protective factors were reported in contributing to more balanced and reflective mental representation, such as positive relationships, e.g., a positive family environment, supportive friendships, and strong partner relationships. This evidence underlines the critical importance of providing comprehensive support both prenatally and postnatally, especially considering the well-documented link between balanced parental representations and secure infant attachment [39,41,46]. It is important to consider that parental mental representations are not static but dynamic and susceptible to change over time, especially as parents engage in direct caregiving experiences, and this aspect should be considered for further analysis of protective and risk factors. In fact, representations may evolve in response to postnatal experiences, such as daily interactions with the infant, co-parenting dynamics, and perceived parental competence. Attention toward both risk and protective factors—as aspects affecting, from a biopsychosocial perspective, both parent and child health—is also supported by the evidence on the neurobiological and psychophysiological correlates of parental representations, suggesting that these internal models are linked to emotional regulation capacities and caregiving-related brain activity. Parents with more coherent and emotionally rich representations may show enhanced activation in brain regions involved in empathy and emotional attunement when interacting with their infant [70]. Therefore, infants protected by a rich and stimulating environment can grow positively and achieve developmental milestones.

Moreover, the representations’ flexibility generally supported by empirical evidence highlights the importance of longitudinal monitoring and context-sensitive interventions, aimed at promoting more adaptive representational styles during the early parenting journey [26]. As the established associations between nonbalanced representations and various psychosocial risk factors, preventive and early intervention strategies should be prioritized during the prenatal period. Results of this systematic summarization suggest the importance of exploring the validity and applicability of programs (e.g., video-feedback interventions or mentalization-based approaches) aimed at developing more reflective, integrated representations of the infant. These interventions could be beneficial for at-risk populations, such as those with a history of trauma, low social support, or elevated psychological distress. Developing and validating tools that can detect changes in parental representations over time is essential to monitor intervention efficacy and personalize support [67].

Another interesting core of this systematic review is the role of reflective functioning (RF) in mental representation. Higher levels of RF are associated with more balanced mental representations. The still few studies on fathers confirmed that moderate-to-high RF correlates with greater preparedness for childbirth and early parenting, increased emotional involvement, and a stronger identification as active co-parents [32,35]. Mothers generally demonstrate higher RF than fathers [56], although the underlying reasons for this gender difference remain unclear due to limited research. While the association between RF and parental representations is generally confirmed by the studies [32,35], the reasons why mothers typically exhibit higher RF than fathers are still poorly understood [56]. A suggestive explanation may be ascribed to the still deep-rooted cultural bias on gender roles in parenthood. Fathers may face structural and emotional barriers to becoming fully engaged co-parents, particularly in contexts where maternal caregiving remains the normative ideal and this aspect, would affect paternal RF and shape mental representations of child and parenting. As a consequence, discrepancies between maternal and paternal representations may impact co-parenting quality, thereby influencing the child’s emotional climate and affecting the family system in unpredictable ways [71]. Studies are needed to explore whether this gender gap is due to biological, psychological, or sociocultural factors, or to differences in caregiving experience and societal expectations. Moreover, longitudinal studies could clarify how RF evolves over time and interacts with contextual factors such as stress, support, and parental mental health. Understanding these developmental trajectories would allow for the more precise identification of at-risk parents and inform the design of tailored interventions aimed at enhancing RF during the perinatal period [67]. Expanding research on RF in diverse populations, especially including both parents and culturally varied samples, would be essential to capturing the complexity of modern parenting and promoting secure early relationships in a range of family contexts.

### Limitations

Despite providing meaningful insights into parental mental representations, this review presents several limitations. First, the heterogeneity of the included studies—particularly in terms of assessment tools and constructs—poses challenges for comparison and synthesis. Various instruments (e.g., WMCI, IRMAG/IRPAG, PAI, PDI-R2) differ in focus, structure, and theoretical underpinnings, limiting the ability to draw unified conclusions about parental representations. Second, most studies focus predominantly on mothers, with paternal representations being under-investigated or explored using non-standardized tools. This creates a gender bias that hinders a comprehensive understanding of how both parents contribute to early relational development. Third, many studies are cross-sectional and lack longitudinal follow-up, preventing the assessment of developmental trajectories of representations and their evolving impact on parent–child interactions. Fourth, several included studies are dated, often reflecting sociocultural contexts that may differ substantially from current family structures and parenting norms. This temporal gap limits the generalizability of findings to today’s diverse parenting scenarios. Finally, few studies adopt a dyadic or systemic lens capable of capturing the interactions between partners and their joint mental representations, despite growing evidence on the relevance of co-parenting dynamics.

## 5. Conclusions

Considering the evidence reviewed, parental mental representations, despite the specific characteristics and differences between mother and father, emerge as a dynamic and multifaceted construct, shaped by individual, relational, and contextual factors. The consistency of associations between balanced representations and parental sensitivity, attachment security, and psychological well-being highlights their central role in early relational development. Moreover, the positive parenting that emerges as related to these representations is confirmed to impact the general health of the child, from physical to mental one across the lifespan [72]. However, the field would benefit from a clearer conceptualization of what constitutes the different expression of representations and how this construct evolves over time. Future studies should adopt longitudinal, dyadic, and interdisciplinary approaches that integrate neurobiological, cognitive–affective, and contextual variables. Moreover, theoretical frameworks such as attachment theory [2], the mentalization-based approach [1,67], and models of co-parenting [73] offer valuable lenses for capturing the complexity of the transition to parenthood, and for this reason further studies must still refer to those classic models to develop new and innovative perspectives. Finally, research should inform early preventive and restorative interventions—especially those that enhance reflective functioning and support vulnerable parents—thus promoting not only individual well-being but also healthier family systems (e.g., [74]). Clinically, the results of this study emphasize the relevance of assessing parental mental representations during the perinatal period to identify early relational risks and guide tailored interventions. The association between nonbalanced representations and psychosocial risk factors highlights the need for preventive programs—such as reflective functioning training or video-feedback interventions—especially among vulnerable populations.

## Figures and Tables

**Table 1:** Script and dataset for systematic search.

Script	Databases
Parent* OR caregiver* OR mother* OR father* AND mental representation* OR reflective functioning	Scopus; APA PSYCARTICLES/APA PSYCINFO; Web of Sciences.

## Data Availability

All the data are reported in this systematic study.
